# Association of High Ratio of CSF/Plasma HIV-1 RNA with Central Nervous System Co-Infection in HIV-1-Positive Treatment-Naive Patients

**DOI:** 10.3390/brainsci12060791

**Published:** 2022-06-16

**Authors:** Qian Liu, Wendan Tao, Honghong Yang, Yushan Wu, Qing Yu, Min Liu

**Affiliations:** 1 Department of Infectious Disease, Chongqing Public Health Medical Center, Chongqing 400036, China; liuqian2199@126.com (Q.L.); yangfengzhuoyu@163.com (H.Y.); wuyushanyouxiang@163.com (Y.W.); yq441702020@sina.com (Q.Y.); 2 Department of Neurology, West China Hospital, Sichuan University, Chengdu 610041, China; taowendan@wchscu.cn

**Keywords:** cerebrospinal fluid, plasma, HIV-1-RNA ratio (HRR), discordance, human immunodeficiency virus, central nervous system, co-infection

## Abstract

Cerebrospinal fluid (CSF) human immunodeficiency virus-1 (HIV-1) ribonucleic acid (RNA) at higher levels than in plasma has been observed in HIV-1-positive patients and defined as CSF/plasma discordance or CSF escape. Discordance is particularly seen in untreated patients with antiretroviral agents. Quantitative data regarding its association with blood–brain barrier (BBB) damage and intracranial co-infection with other pathogens are limited. Therefore, we used the CSF to plasma HIV-1 RNA ratio (HRR) to determine its relation to central nervous system (CNS) co-infection in HIV-1-positive treatment-naïve individuals. We retrospectively recruited the subjects with HIV-1-positive and potential neurological deficits. A lumbar puncture was performed before the antiretroviral therapy. The paired CSF/plasma HIV-1 RNA samples were analyzed. Univariate and multivariate logistic regression models and multiple spine regression analyses were performed to assess the association between the HRR and CNS co-infection. A total of 195 patients with 78% males (median age: 49 years) were included in this study, of whom 98 (50.2%) had CNS co-infection with other pathogens. The receiver-operating characteristic curve analysis showed that the optimal cutoff value for the HRR to predict the CNS co-infection was 1.00. Higher HRR (≥1) was significantly associated with tuberculous meningitis (OR 6.50, 95% CI 2.08–20.25, *p* = 0.001), cryptococcus meningitis (OR 7.58, 95% CI 2.10–27.32, *p* = 0.001), and multiple co-infection (OR 4.04, 95% CI 1.02–16.04, *p* = 0.047). Higher HRR (≥1) (OR 3.01, 95% CI 1.09–8.73, *p* = 0.032) was independently associated with the CNS co-infection after adjusting for covariates. No significant nonlinear association was found between the HRR and CNS co-infection in the multivariate spline regression (*p* > 0.05) and a positive relationship was found between the HRR and CNS co-infection when the HRR was ≥0.78. Higher HRR was associated with an increased risk of CNS co-infection in HIV-1-positive patients. The relationship between the HRR and CNS co-infection may be related to the BBB disturbance and warrants further investigation with a large, longitudinal cohort.

## 1. Introduction

The central nervous system (CNS) is vulnerable to be affected in patients with positive-human immunodeficiency virus-1 (HIV-1) [1,2,3]. The HIV-1 virus enters the cerebrospinal fluid (CSF) through blood, especially via the choroid plexus, via the trafficking of infected peripheral blood lymphocytes or monocytes, and infects the meninges or brain [1,4]. The HIV-1 RNA in plasma or CSF is an important prognostic marker of disease progression [5,6,7].

Generally, the integrity of the BBB maintains the viral load, and thus HIV-1 RNA, in plasma higher than CSF [5]. However, previous studies reported 5%–32% of individuals with higher levels of HIV-1 RNA in the CSF than the plasma, and this is referred to as CSF/plasma discordance or CSF escape [8,9,10,11]. The discordance occurs in individuals who never receive antiretroviral treatment (ART), whereas the escape happens in individuals who receive antiviral treatment [12,13]. The occurrence rate of discordance or escape might be inaccurate, since the analyses usually include patients receiving and not receiving antiretroviral treatment. The level of HIV-1 RNA in the CSF or plasma to some extent is suppressed by the antiretroviral drugs (ARVs) [14]. It is difficult to explain whether the discordance/escape phenomenon is due to the problem of the BBB permeability to the ARVs, which results in a slow decline of the CNS HIV-1 RNA than the blood HIV-1 RNA, or independent replication and evolution of the HIV-1 within the CNS [15]. The HIV-1 RNA variation in the CSF/plasma in antiretroviral treatment-naïve patients need to be investigated and its associated risk factors need to be identified.

A proportion of HIV-1-positive treatment-naive patients (HPTNPs) shows neurological symptoms. HIV-1 could attack the immune cells and frequently compromise the integrity of the BBB. The BBB impairment is common in HIV-1-positive patients, which increases the risk of opportunistic co-infection in the central nervous system, such as tuberculous meningitis and cryptococcal meningitis [16,17,18]. Those co-infections often happen in patients who did not receive medical care in early stages and are the major cause of morbidity and mortality in HIV-1-positive individuals [18,19]. Furthermore, CNS co-infection with the above pathogens leads to a severe inflammatory reaction in the CNS, affects the internal environment, and may accelerate the replication of the HIV-1 RNA in the CNS [20,21]. Rapid and continuous HIV-1 replication in the CNS leads to viral neurotoxicity, chronic sustained immune activation, and evolution of drug-resistant CNS HIV-1, which results in acute or subacute neurological symptoms and deteriorates the prognosis [22]. We hypothesize that the HIV-1 RNA discordance is associated with the BBB damage, and thus is correlated with the intracranial co-infection.

The CSF/plasma ratio of HIV-1-RNA (HRR) of >1 was labeled as a discordance in a few previous studies, but little is known about the effects of the HRR on the AIDS complicated with the CNS co-infection [23]. Therefore, we aimed to use the HRR to determine the dynamic changes of HIV-1 RNA in the CSF and plasma and justify whether the HRR is potentially correlated with the CNS co-infection in HPTNP.

## 2. Materials and Methods

### 2.1. Study Design

This is a cross-sectional study which retrospectively collected data of HIV-1 infected patients who met the diagnostic criteria and admitted to the Chongqing public health medical center from January 2020 to June 2020 for this study. The HIV-1-positive diagnosis was in accordance with the European AIDS Clinical Society (EACS) Guidelines 2020 [24]. All the patients included met the following criteria: (1) aged >18 years old; (2) never had ART previously; (3) with neurological symptoms such as walking imbalance, memory and speech disturbance, headache, pain and sensory disruption, and seizures or intracranial lesions on MRI/CT, or hematogenous dissemination of TB with unconfirmed intracranial tuberculosis. The study was approved by the Chongqing Public Health Medical Center Institutional Ethics Committee (approval code: 2021-009-01-KY). The data are anonymous and retrospectively included; therefore, the requirement for informed consent was waived.

### 2.2. Data Collection

The detailed clinical data such as demographic information, laboratory tests results, and different types of CNS co-infection were collected for analysis.

### 2.3. Laboratory Assessments

HIV-1 RNA detection: 10 mL of venous blood was collected using an EDTA anticoagulant tube, centrifuged to separate the plasma, and then frozen to −80 ℃ in an ultra-freezer. The plasma HIV-1-RNA level was quantitatively detected using an automatic real-time fluorescence quantitative detector (COBAS Taq-MAN48, Roche Diagnostics, Germany). The lowest detection down-line value was 20 copies/mL.

The CD4+T lymphocyte detection: A 2 mL venous blood was collected by using an EDTA anticoagulant tube, and the number of CD4+T cells in the samples was analyzed by using a Trucount absolute counting tube in a flow cytometer (BD Biosciences, Franklin Lakes, NJ, USA). The cells in the CSF were counted and classified using SYSMEX XE-4000 (Shanghai, China). The CSF protein and glucose were detected using colorimetry, and the CSF chloride was detected using the direct electrode method. All tests are carried out in the Chongqing public health medical center clinical laboratory.

### 2.4. CNS Co-Infection Assessments

The CNS co-infection was identified from the discharge medical records. Diagnosis criteria were as follows [25,26,27]:

1. Tuberculous meningitis: (i) The CSF with lymphocytic pleocytosis, low glucose, and elevated protein; (ii) the brain CT/MRI showing enhancement of the meninges and the periphery with the tuberculoma lesions; (iii) the culture or CSF smear or CSF-PCR; (iv) the successful response by a specific treatment. The diagnosis complied with (iii) or any two of the other three criteria.
2. Toxoplasma meningitis should have been consistent at the same time: (i) progressive neurological deficits; (ii) contrast-enhancing mass lesion(s) on the CT/MRI; (iii) successful response within 2 weeks of the specific treatment.
3. Cryptococcal meningitis needed to meet any one of these criteria: (i) visualizing the fungus in the CSF using India ink; (ii) detecting the cryptococcal antigen by the latex agglutination assay in the CSF; (iii) positive CSF culture for C. neoformans.
4. Diagnosis of neurosyphilis should have been consistent at the same time: (i) epidemiological history; (ii) clinical manifestations; (iii) positive serum TPPA; (iv) CSF leukocyte count of ≥20 × 10^6^/L; CSF protein of ≥500 mg/L; (v) CSF TPPA positive.
5. Viral encephalitis included CMV (cytomegalovirus) and JC virus (JCV). Diagnosis of CMV encephalitis via clinical appearance, positive PCR in CSF, and other pathology was excluded. Diagnosis of Progressive Multifocal Leukoencephalopathy, which is caused by JCV, depends on the evidence of JCV-deoxyribonucleic acid (JCV-DNA) in CSF and compatible clinical-radiological picture.
6. Multiple co-infections referred to the combination of more than two types of co-infection
7. Detection of co-infectious pathogens mentioned in our manuscript by Next-Generation Sequencing (NGS) testing.

### 2.5. Statistical Methods

The continuous variables were tested for normality at first. The mean ± standard deviation were used to describe the continuous variables with the normal distribution. The median with the interquartile range was used to describe variables with non-normal distribution. The percentages were used to describe the categorical variables. The chi-square test and the Mann–Whitney U test were performed for the comparisons of variables between different subgroups as appropriate.

The receiver operating characteristics (ROC) curve analysis was used to identify the optimal cut-off value for the continuous HRR to predict the ratio of the CSF/plasma. Then, the HRR was dichotomized in accordance with its optimal cutoff value and tested using a multivariate logistic regression model. The univariate and multivariate logistic regression models were adopted to examine the association between the HRR and the CNS co-infection group. The variables with the *p* < 0.1 in the univariate analysis were considered to be included in the multivariate logistic regression models. The odds ratios (ORs), as well as the corresponding 95% confidence interval (CI) values, were reported. The restricted cubic splines were used to examine the shape of the association between the X and Y with three knots (10th, 50th, and 90th percentiles).

All the analyses were performed with the SPSS 19.0 (IBM, Chicago, IL, USA) and Stata 15.0 (Stata Corp LP, College Station, TX, USA) software. The *p*-values less than 0.05 were considered as a significant difference.

## 3. Results

A total of 195 (mean age, 48.8 years; 77.9% males) HPTNPs were included in this study, of whom 98 (50.2%) were identified as having CNS co-infection. Among the patients with CNS co-infection, 30 (30.6%) manifested tuberculous meningitis (TBM), 18 (18.3%) manifested cryptococcus meningitis (CM), 14 (14.2%) manifested viral encephalitis (VE), 11 (11.2%) exhibited toxoplasma meningitis (TM), 6 (6.1%) showed neurosyphilis (NS), and 19 (19.3%) showed multiple co-infection (MI).

The baseline characteristics are presented in Table 1. The patients with the CNS co-infection had a higher level of HIV-1 RNA in the CSF and a higher CSF/plasmaHRR. Further, the CSF protein and CSF white blood cells were greater compared with those without CNS co-infection (All *p* < 0.001).

**Figure 1:**
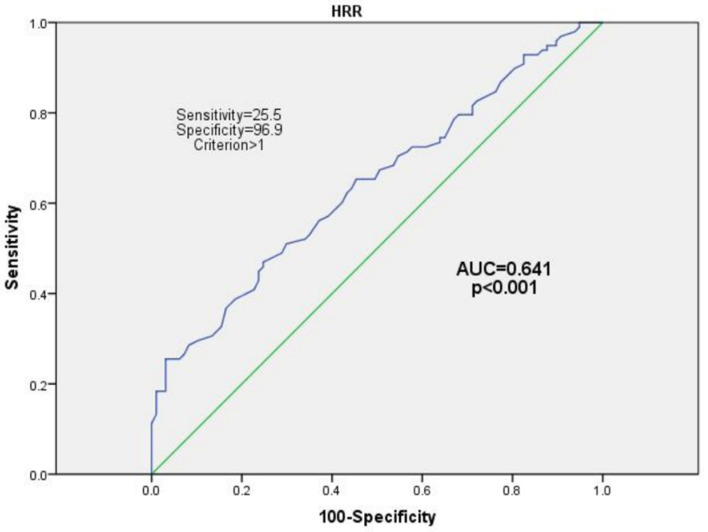
The optimal cutoff value to evaluate the association between CSF/plasma HRR and CNS infection was 1 [AUC 0.64; 95% CI (0.57–0.70); *p* < 0.001]. CSF/plasma HRR WAS dichotomized in accordance with its optimal cutoff value (1.00) and tested in multivariate logistic regression models.

Based on the ROC curve, the optimal cutoff value of the HRR to predict the CNS co-infection was 1.00 [AUC 0.64; 95% CI (0.57–0.70); *p* < 0.001] (Figure 1). The HRR was dichotomized following its optimal cutoff value (1.00) and tested using the multivariate logistic regression model.

Table 2 demonstrates the univariate analyses between the HRR and CNS co-infection. The higher HRR (≥1) was significantly associated with CNS co-infection (OR, 5.19, 95% CI, 2.02–13.33, *p* = 0.001), tuberculous meningitis (OR, 6.50, 95% CI, 2.08–20.25, *p* = 0.001), cryptococcus meningitis (OR, 7.58, 95% CI, 2.10–27.32, *p* = 0.001), and multiple co-infection (OR, 4.04, 95% CI, 1.02–16.04, *p* = 0.047).

### Multivariate Analyses between the HRR and CNS Co-Infection

**Figure 2:**
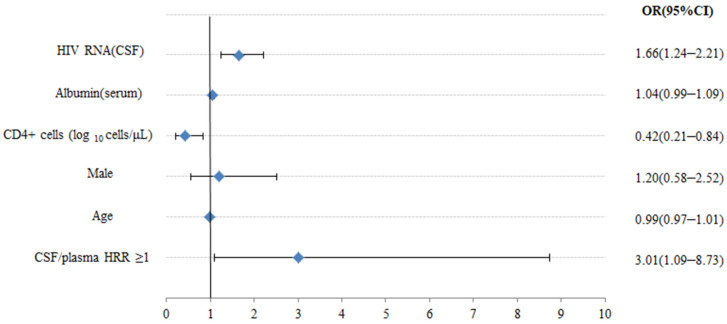
Multivariate logistic regression analysis between clinical features and CNS co-infection. OR—odds ratio; CI—confidence level.

Figure 2 illustrates the multivariate analyses between the HRR and CNS co-infection. After adjusting for the age, sex, serum albumin, and CD4+ cell count, we still detected that higher CSF/plasma HRR (≥1) (OR 3.01, 95% CI 1.09–8.73, *p* = 0.032), higher HIV-1 RNA in CSF (OR 1.66, 95% CI 1.24–2.21, *p* = 0.001), and lower CD4+ cell count (OR 0.42, 95% CI 0.21–0.84, *p* = 0.014) were significantly associated with increased risk of CNS co-infection. Furthermore, we adopted HRR levels in the model as continuous data after potential confounders were adjusted in Figure 2, and no significant nonlinear association was found between HRR and CNS co-infection in the multivariate spline regression (*p* > 0.05), suggesting a linear relationship between the HRR and CNS co-infection. In addition, a positive relationship was found between the HRR and CNS co-infection when the HRR was ≥0.78.

## 4. Discussion

In our cohort, we provided preliminary evidence about the relationship between HRR and CNS co-infection in HIV-1 positive treatment-naïve patients. Due to the different research objectives, the definitions of discordance/escape have yielded inconclusive standards [12]. Rawson [28] and Nightingale [10] defined it as the CSF HIV-1 RNA of more than 0.5 log10 copies/mL and higher than plasma level or the CSF HIV-1 RNA of >20 copies/mL with an undetectable plasma RNA, whereas Canestri [8] defined it by any detectable CSF HIV-1 RNA level of >200 copies/mL with the plasma levels of <50 copies/mL or by a CSF HIV-1 RNA level of >1 log and higher than the plasma level. Thus, we searched for a simple way to address the inconsistency: the CSF/Plasma HIV-1 RNA ratio. In addition, most current studies included patients that have been receiving combination ART, which was considered an important confounder when evaluating the inconsistency. Since the variable BBB permeability among different ART, CSF HIV-1 RNA escape could occur in patients receiving ART, which could not effectively inhibit HIV-1 replication in CSF [29,30]. The present study introduced the concept of the HRR that was first proposed by Merlini in 2018 and regarded as HRR of ≥1 in paired CSF/Plasma samples in the treatment-naive patients as a simple cutoff and optimum indicator to define the discordance [23]. We also found the HRR of ≥1 with a high specificity but low sensitivity, which indicated the HRR of ≥1 considered as CNS co-infection occurred less often than was expected.

Investigating the mechanisms and risk factors associated with CSF/plasma HIV-1 discordance is useful for choosing an appropriate systemic intervention. This study contributes to the emerging literature and provides further evidence that higher HRR was independently associated with an increased risk of CNS co-infection in HPTNPs. Although there have been several previous studies about discordance, they often described the poor patient prognosis. Anderson AM revealed that high CSF HIV-1 RNA was correlated with active CNS viral replication in the HIV-1-associated neurocognitive disorder (HAND) [31,32]. Dravid found that the prevalence of CSF/plasma HIV-1 RNA discordance was higher among neurologically symptomatic patients with hypo-viremia, which means plasma VL was between 20 and 1000 copies/mL [9]. However, there were few studies exploring the risk factors of high HRR in HIV-1-infected patients before the antiviral treatment. It was unclear why higher HRR occurred and which patients were more prone to high HRR. We found that higher HRR was significantly associated with an increased risk of CNS co-infection, especially with some specific pathogen co-infection. The HRR of ≥1 was significantly associated with tuberculous meningitis and cryptococcus meningitis. Therefore, HPTNPs and the HRR of ≥1 might need to be vigilant towards the occurrence of CNS co-infection. Additionally, whether dynamic monitoring of HRR can be used as a better indicator to evaluate the efficacy and prognosis of ART still needs further research.

In addition to the higher HRR being associated with the CNS co-infection, we also observed that the higher absolute value of the HIV-1 RNA in the CSF and low CD4+ cell counts were also associated with the CNS co-infection in the HIV-1-infected patients; the result is consistent with another study [6]. At the same time, research revealed that the CSF white blood-cell count was effective as a predictive biomarker of CSF and plasma discordance [33]. Therefore, for patients with high CSF HIV-1 RNA, it is necessary to consider the blood–brain permeable antiviral drugs with higher CNS penetration efficiency (CPE) scores, based on the six categories classified by the United States Food and Drug Administration (US FDA), the commonly used ARVs such as emtricitabine, dolutegravir, darunavir/r, etc. [29,34,35]. The issue of which strategy is best suited for the treatment and prevention of CNS co-infection is still under debate. Conventional wisdom considers the penetration of antiviral substances into the CSF and brain parenchyma as an important role. Most studies [14,27,36] showed associations between higher CPE scores and lower CSF viral loads. Canestri reported that after ART optimization based on the CPE, all patients with discordance between CSF and plasma HIV-1 RNA and neurological symptoms had clinical improvement [8]. Ferretti. F [37] found that CSF escape in most of the cases which followed of ART simplification towards regimens that were associated with drug resistance or low neuro-penetration and efficacy. Therefore, based on these studies, it is possible to suggest strengthening ART when patients have high CSF HIV-1 RNA or when the HRR is ≥1. Therefore, routine evaluation of the CSF/plasma HIV-1 RNA, inflammatory indicators of CSF, and CD4+ cells counts in the patients with neurological symptoms is recommended to guide further treatment. However, ART drugs with high CPE can effectively inhibit HIV-1 replication in CSF for longer periods, which may result in neurotoxicity, including emotional disturbance, insomnia, and peripheral neuropathy [30,38]. Therefore, it is particularly important to determine the appropriate plasma and CSF concentration of ART to avoid neurotoxicity. Future studies should quantify the ART therapeutic ranges in the CNS, not only to determine which antiretroviral drugs penetrate the CNS adequately to suppress viral replication, but also which antiretroviral drugs penetrate the CNS to an extent that they contribute to neurotoxicity. The guidelines recommend that minimal toxicity, low pill burden, and simplified therapy in ART also show an important role in plasma viral control [39].

The present study had several limitations. First, we did not measure the dynamic changes of the HRR before and after the ART. Whether the reduced HRR is associated with CNS co-infection improvement needs further investigation. Second, although we adjusted the cases with CNS co-infection, due to the small sample size, we could not conduct the subgroup multivariate analyses. Third, due to the cross-sectional design, we could not assess the causation. Further longitudinal studies are needed to assess its effect for long-term outcomes. To the best of our knowledge, our study is one of the reports of a large number of neuro-symptomatic CSF/plasma HIV-1 discordance cases. Larger sample size and longitudinal follow-up are needed to further clarify the value of the HRR in the clinical diagnosis and treatment.

## Figures and Tables

**Table 1:** Baseline characteristics according to central nervous co-infection.

	CNI Group (*N* = 98)	Non-CNI Group (*N* = 97)	*p*-Value
Age (mean ± SD)	46.74 ± 12.65	49.74 ± 16.15	0.239
Male gender, n (%)	76(77.55)	75(77.32)	0.969
**Disease characteristics**			
CD 4 (cell per µL), median (IQR)	20 (39–72)	41 (19–106)	0.365
Ratio of CD4/CD8, median (IQR)	0.08 (0.13–0.20)	0.145 (0.08–0.24)	0.326
Serum albumin (mean ± SD)	35.63 ± 6.84	33.97 ± 6.38	0.072
Plasma HIV RNA (Log copies/mL), median (IQR)	5.86 (5.50–6.27)	5.69 (5.19–6.19)	0.158
CSF HIV RNA (log copies/mL), median (IQR)	4.78 (3.67–5.55)	3.98 (3.10–4.65)	<0.001
CSF/plasma HIV RNA ratio, (mean ± SD)	0.82 ± 0.22	0.71 ± 0.19	<0.001
CSF protein (mg/L), median (IQR)	594.86 (362.96–978.64)	359.01 (310.93–451.22)	<0.001
CSF chloride (mmol/L), mean ± SD	121.0 ± 8.75	124.42 ± 4.36	0.001
CSF glucose, (mmol/L), median (IQR)	3.19 (2.44–3.60)	3.66 (3.23–4.33)	<0.001
CSF white blood cells (cell per µL), median (IQR)	5 (0–84)	1 (0–4)	<0.001

CNI-group: CNS co-infection group; non CNI-group: non-CNS co-infection group; univariate analyses to assess the association between the HRR and CNS co-infection subtypes.

**Table 2:** Univariate analyses to identify risk factors for different types of central nervous co-infection.

Variables	CNS Co-Infection (*N* = 98)		TBM (*N* = 30)		CM (*N* = 18)		TM (*N* = 11)		VE (*N* = 14)		NS (*N* = 6)		MI (*N* = 19)	
	OR (95% CI)	*p* Value	OR (95% CI)	*p* Value	OR (95% CI)	*p* Value	OR (95% CI)	*p* Value	OR (95% CI)	*p* Value	OR (95% CI)	*p* Value	OR (95% CI)	*p* Value
CSF/plasma HRR														
<1.00														
≥1.00	5.19 (2.02–13.33)	0.001	6.50 (2.08–20.25)	0.001	7.58 (2.10–27.32)	0.002	3.37 (0.59–19.21)	0.171	4.13 (0.90–18.92)	0.067	3.03 (0.30–30.27)	0.344	4.04 (1.02–16.04)	0.047
CSF/plasma HRR	13.78 (3.27–58.13)	<0.001	8.92 (1.11–71.19)	0.039	NA	NA	NA	NA	NA	NA	NA	NA	NA	NA
Age	0.98 (0.96–1.00)	0.153	0.98 (0.96–1.01)	0.348	0.98 (0.95–1.02)	0.478	0.99 (0.95–1.03)	0.785	0.99 (0.96–1.03)	0.855	0.94 (0.88–1.00)	0.073	0.99 (0.95–1.02)	0.532
Male	0.98 (0.50–1.93)	0.969	1.24 (0.48–3.16)	0.654	1.31 (0.42–4.08)	0.640	1.27 (0.31–5.23)	0.733	0.93 (0.23–3.63)	0.917	NA	NA	0.63 (0.17–2.39)	0.507
CD 4 count (log _10_ cell per µL)	0.71 (0.39–1.28)	0.262	0.79 (0.35–1.77)	0.572	0.47 (0.17–1.36)	0.165	0.76 (0.21–2.78)	0.683	1.21 (0.39–3.76)	0.738	1.89 (0.32–10.85)	0.475	0.44 (0.15–1.28)	0.133
Ratio of CD4/CD8	0.25 (0.03–1.79)	0.170	0.30 (0.01–5.32)	0.418	0.00 (0.00–0.98)	0.050	0.03 (0.00–9.13)	0.234	1.12 (0.03–35.72)	0.947	NA	NA	0.89 (0.03–20.61)	0.946
Serum albumin	1.03 (0.99–1.08)	0.087	1.02 (0.95–1.08)	0.550	1.04 (0.97–1.11)	0.183	1.04 (0.94–1.15)	0.389	1.04 (0.96–1.14)	0.281	1.06 (0.93–1.21)	0.324	1.04 (0.96–1.13)	0.304
Plasma HIV RNA (Log copies/mL)	1.25 (0.90–1.73)	0.167	1.25 (0.78–1.99)	0.346	1.79 (0.88–3.64)	0.103	1.225 (0.63–2.39)	0.551	0.87 (0.48–1.58)	0.666	0.65 (0.30–1.42)	0.284	1.89 (0.93–3.84)	0.076
CSF HIV RNA concentration (Log copies/mL)	1.71 (1.32–2.20)	<0.001	1.59 (1.11–2.29)	0.011	2.92 (1.67–5.10)	<0.001	1.55 (0.84–2.87)	0.158	1.34 (0.79–2.26)	0.266	1.34 (0.61–2.92)	0.457	2.27 (1.36–3.79)	0.002
CSF protein (mg/L)	1.003 (1.002–1.004)	<0.001	1.003 (1.001–1.004)	<0.001	1.002 (1.001–1.004)	0.006	1.002 (1.002–1.004)	0.014	1.002 (1.000–1.004)	0.027	1.003 (1.000–1.005)	0.021	1.003 (1.001–1.004)	0.001
CSF chloride(mmol/L)	0.92 (0.88–0.97)	<0.001	0.91 (0.84–0.97)	0.011	0.86 (0.78–0.96)	0.009	0.96 (0.83–1.10)	0.574	0.93 (0.82–1.05)	0.271	0.96 (0.79–1.17)	0.744	0.86 (0.79–0.94)	0.001
CSF glucose(mmol/L)	0.62 (0.47–0.82)	0.001	0.59 (0.38–0.89)	0.013	0.31 (0.16–0.61)	0.001	0.29 (0.10–0.87)	0.027	0.54 (0.25–1.17)	0.122	0.18 (0.04–0.66)	0.010	0.74 (0.45–1.22)	0.249
CSF white blood cells (cell per µL)	1.05 (1.01–1.09)	0.005	1.05 (0.99–1.10)	0.061	1.10 (1.03–1.18)	0.005	1.08 (0.99–1.17)	0.053	1.04 (1.00–1.08)	0.035	1.05 (0.99–1.11)	0.069	1.07 (1.00–1.14)	0.039

HRR—HIV RNA ratio; TBM—Tuberculous meningitis; CM—Cryptococcus meningitis; VE—viral encephalitis; TM—Toxoplasma meningitis; NS—neurosyphilis; MI—multiple co-infection.

## Data Availability

Data are not publicly available due to privacy reasons of patients, but the data presented in this study are available on request from the corresponding author.
